# Editorial: Microbiota-immune interactions: a new frontier in cancer treatment optimization

**DOI:** 10.3389/fimmu.2026.1882251

**Published:** 2026-06-03

**Authors:** Stephen Blake, Lorenzo Mortara, Sheila Spada

**Affiliations:** 1 Precision Cancer Medicine Theme, South Australian Health and Medical Research Institute, Adelaide, SA, Australia; 2 Flinders Health and Medical Research Institute, Flinders University, Bedford Park, SA, Australia; 3 Immunology and General Pathology Laboratory, Department of Biotechnology and Life Sciences, University of Insubria, Varese, Italy; 4 Tumor Immunology and Immunotherapy Unit, IRCCS-Regina Elena National Cancer Institute, Rome, Italy

**Keywords:** cancer, dysbiosis, immunotherapy, metabolism, microbiome, transplantation

Microbiome research has expanded rapidly over the past decade, substantially advancing our understanding of how microbial communities contribute to different diseases, including cancer (1–3). Perturbations of specific microbial communities, referred to as dysbiosis, can induce profound metabolic reprogramming and immune dysregulation within the host. These microbiota-driven alterations influence numerous immunological pathways, altering how innate and adaptive immune cells function and potently reshaping localized and systemic immune responses. Despite recent advances, the precise mechanistic pathways through which microbiota-immune interactions lead to tumor development and progression, along with the impact of anti-tumor therapeutic response on tumor control, remain largely undefined. There also remains a major challenge in translating microbiota research into routine clinical practice. The high inter-individual variability of microbial ecosystems, influenced by genetics, diet, environment, and prior therapies, complicates the identification of universally applicable microbial signatures, necessitating longitudinal studies and mechanistic validation in well-defined preclinical models. Standardization of microbiome profiling methods, analytical pipelines, and interventional strategies is also urgently required to ensure reproducibility and clinical translatability across studies.

The Research Topic *“Microbiota-Immune Interactions: A New Frontier in Cancer Treatment Optimization”* comprehensively illustrates both the perspectives and the challenges of this rapidly evolving field. It comprises 6 original articles, 7 review articles, and 1 systematic review article.

The involvement of the microbiome in gynecological cancers and its clinical implications are largely underexplored. Oyedokun et al. reviewed dysbiosis in the vaginal, endometrial, ovarian, and gut microbiomes with the depletion of beneficial Lactobacillus species and the overrepresentation of anaerobic organisms such as *Fusobacterium*, *Atopobium*, and *Sneathia*. These alterations lead to chronic inflammation, an imbalance in immune response, and estrogen metabolism. They also promote DNA dysfunction and abnormal cell growth, resulting in gynecological cancers. The authors highlight the potential of the microbiome and its microbial signature as non-invasive biomarkers and as potential areas of intervention that include the use of pre- and probiotics, dietary modifications, and, when possible, microbiota transplantation.

Similarly, the narrative review by Chen et al. highlighted the impact of the microbiota-reproductive axis, including gut and vaginal microbes, in modulating the progression of and response to therapy for gynecological malignancies. The microbiome, described as an endocrine organ named the “estrobolome”, regulates the systemic recirculation of estrogens and shapes a hyperestrogenic microenvironment that fuels hormone-dependent tumorigenesis. Moreover, the microbiota controls the balance between immunosurveillance and inflammation to sustain mucosal barrier integrity and favor tolerance. Conversely, dysbiosis triggers chronic inflammation, which drives immunosuppression. Moreover, pathobionts induce genotoxicity, which generates genomic instability, a hallmark of cancer. From a translational point of view, the authors also discuss the use of probiotics, fecal microbiota transplantation, and precision microbial therapies as innovative approaches for the diagnosis, prognosis, and treatment of cancer.

The article by Dora et al. investigated the role of proteomes in urinary extracellular vesicles (EVs) derived from both the host and microbiota, as non-invasive predictive biomarkers for progression-free survival (PFS) in patients with advanced non-small cell lung cancer (NSCLC) treated with anti-PD1 inhibitors. Specifically, bacterial proteins in urinary EVs were found to correlate with gut microbial abundance and PFS, revealing immune-microbiota interactions, with a higher bacterial/host protein ratio that correlates to a longer PFS. The authors integrated proteomic data with machine learning to identify predictive protein signatures that could inform personalized immunotherapy approaches. Preclinical and clinical studies linking gut microbiota composition to immune checkpoint blockade (ICB) outcomes in NSCLC were outlined in the systematic review from Jin et al. The authors identified that the enrichment of the *Actinobacteria*, *Bacteroidetes*, and *Verrucomicrobia* phyla, along with higher microbial diversity, are associated with better ICB efficacy and reduced immune-related adverse events. They also identified species, including *Bifidobacterium* in mice, that enhance ICB efficacy via improved antigen presentation, T-2011; cell priming, and migration. Microbiota-altering treatments, including exposure to antibiotics or proton pump inhibitors 1 month prior to ICB, were found to be detrimental in the majority of studies, while a smaller number of studies found that probiotics and diet were associated with increased microbiota diversity and composition and ICB efficacy.

Also investigating lung cancer, the clinical correlative study by Song et al. evaluated the microbiota composition and fecal metabolites of 26 patients with extensive-stage small cell lung cancer (ES-SCLC) who were treated with combined chemotherapy and ICB. Separating responders/non-responders by progression-free survival duration, the authors performed metagenome sequencing and metabolite quantification on stool samples collected before and during treatment, identifying numerous correlations between specific gut microbiota, metabolite production, and response to treatment. While a small cohort was analyzed, the authors’ findings support the use of the gut microbiome as a predictive biomarker for ES-SCLC patients treated with chemoimmunotherapy.

The effect of urinary tract and gut microbiota on the prognosis and immunotherapy response of patients with bladder, renal, and prostate cancer was also investigated in a systematic review by Zou et al. Importantly, the research group focused on the spatial localization of the microbiome and how this influences the molecular mechanisms of anti-tumor immunity that reflect therapy response. A better understanding of the role of the microbiome will pave the way for new cancer prevention strategies and therapeutic approaches.

The article by Tajpara et al. explored the oncogenic properties of microbes isolated from patients with intraductal papillary mucinous neoplasms (IPMNs). The authors took advantage of pancreatic spheroid models to mimic the tumor microenvironment and demonstrated that patient-derived microbes are able to colonize healthy spheroids in an oxygen-dependent manner and induce double-stranded DNA damage in pancreatic spheroids. Notably, they showed that the microbes are recognized and activate peripheral mucosal-associated invariant T (MAIT) cells via riboflavin-derived metabolite sensing, with potential clinical implications for cancer management.

The review from Yang et al. outlined the mechanisms by which gut microbiota shape immune responses during hepatocellular carcinoma (HCC) development and treatment. The authors outlined the bidirectional gut–liver axis and described the microbial dysbiosis observed in patients with HCC. Key microbial metabolites, including endotoxins, secondary bile acids, short-chain fatty acids, and indoles, along with their contribution to the profoundly immunosuppressive tumor microenvironment of HCC, are described, as is the potential of targeting the microbiome to improve ICB outcomes in HCC. Another interesting article review on the interconnection between the microbiota and HCC was presented by Chen P. et al. In their work, the authors analyzed the role of modulation of the intestinal microbiota through probiotics and how this can affect the efficacy of cancer immunotherapies. Gut microbiota dysbiosis could play a central role in HCC by generating carcinogenic products, altering the equilibrium between the immune system and the microbiota, and increasing intestinal barrier permeability. This study emphasizes and describes the mechanisms by which probiotics regulate metabolic processes, inflammation, and immune responses that are relevant to boosting antitumor immune responses.

The review from Tingting et al. focused on the role of the intratumoral microbiota in shaping the tumor immune microenvironment. The authors outlined key intratumoral bacterial and fungal species, along with the cancers they are known to colonize, while also discussing how these alter the tumor immune environment to influence cancer progression. The authors summarized the bispecific role that intratumoral bacteria have been shown to play in both inhibiting and enhancing the efficacy of different cancer therapies. The methodological challenges in studying intratumoral microbes were also discussed, as were the clinical potential of tumor microbiota as a cancer diagnostic tool and a target to enhance responses to immunotherapy.

The review from Pan et al. explored the involvement of the infection–microbiome–immunity axis in the carcinogenesis of bladder cancer. The authors identified and discussed different urinary infectious agents in combination with immune dysfunction. They also illustrated how persistent inflammation, oxidative stress, and epithelial injury are able to contribute to urothelial carcinogenesis. At the same time, the authors analyzed the phenomenon of senescence and its role in worsening microbial persistence and inhibiting the anti-tumor immune response. Finally, they discussed innovative, microbiome-targeted, and immunomodulatory strategies aimed at restoring microbial–immune equilibrium and improving therapeutic efficacy.

Conversely, Hirasawa et al. investigated the relationship between the aging of the gut microbiota and the efficacy of ICB by studying stool samples from 36 elderly patients with various solid tumors before the start of immunotherapy. In the literature, data on age-related changes in the gut microbiota of patients with cancer are largely unexplored. The analysis of gut microbial composition through next-generation sequencing revealed that in patients aged ≥75 years, the presence of *Veillonella* species significantly decreased, whereas *Streptococcus* species was relatively more abundant. However, this study is still observational with a limited sample size and lacks a mechanism for the relationship between aging and ICB efficacy. Therefore, further studies with larger cohorts are needed.

Finally, the study by Zhou et al. analyzed the relationship between gut fungal biomarkers and bacterial-fungal interactions in three cohorts of cutaneous melanoma (CM) patients. In this experimental work, the authors employed metagenomic sequencing analysis of stool samples collected prior to immunotherapy. Biomarkers and key bacterial-fungal interactions were determined via metagenomics, machine learning, and SHapley Additive exPlanations (SHAP) methodology. This study revealed changes in gut bacterial and fungal composition in CM responders. Of note, analysis of bacterial-fungal interactions revealed a positive correlation between *Akkermansia* and *Rutstroemia* in all cohorts, which may be a key element associated with immunotherapy response in CM patients.

Overall, this Research Topic underscores the microbiota–immune axis as a new frontier in cancer research and treatment optimization. Integrating multi-omics approaches, including metagenomics, metabolomics, and transcriptomics, provides a powerful framework for decoding the functional impact of the microbiota on antitumor immunity, paving the way for microbiome-based interventions and more personalized, effective therapeutic strategies that can enhance the efficacy and durability of immunotherapy for patients with cancer.
